# Homography Ranking Based on Multiple Groups of Point Correspondences

**DOI:** 10.3390/s21175752

**Published:** 2021-08-26

**Authors:** Milan Ondrašovič, Peter Tarábek

**Affiliations:** Faculty of Management Science and Informatics, University of Žilina, Univerzitná 8215/1, 010 26 Žilina, Slovakia; peter.tarabek@fri.uniza.sk

**Keywords:** homography matrix, many-to-one point correspondence, perspective distortion, ranking method, bird’s-eye view

## Abstract

Homography mapping is often exploited to remove perspective distortion in images and can be estimated using point correspondences of a known object (marker). We focus on scenarios with multiple markers placed on the same plane if their relative positions in the world are unknown, causing an indeterminate point correspondence. Existing approaches may only estimate an isolated homography for each marker and cannot determine which homography achieves the best reprojection over the entire image. We thus propose a method to rank isolated homographies obtained from multiple distinct markers to select the best homography. This method extends existing approaches in the post-processing stage, provided that the point correspondences are available and that the markers differ only by similarity transformation after rectification. We demonstrate the robustness of our method using a synthetic dataset and show an approximately 60% relative improvement over the random selection strategy based on the homography estimation from the OpenCV library.

## 1. Introduction

Homography is a perspective projection of a plane from one camera view into a different camera view. The perspective projection maps points from a 3D world onto a 2D image plane along lines that emanate from a single point [1,2]. This projection is performed by a 3×3 invertible transformation matrix called the homography matrix (or just homography) with eight degrees of freedom (DoF). In the pinhole camera model, any two images of the same planar surface are related to each other by the homography [3,4]. Homography is commonly used for the rectification of text document images by generating a fronto-parallel view [5,6], image stitching [7,8], video stabilization [9], extracting metric information from 2D images [10], and pose estimation [11] and for various traffic-related applications, e.g., ground-plane detection [12] and bird’s-eye view projection [13].

Homography estimation is essential for image registration, i.e., a process of image matching and transformation of two or more different images [14]. It can be addressed either on the pixel or feature levels. In our work, we focus on feature-based approaches that utilize only a subset of pixels. A common approach to estimating the homography is to use a set of at least four 2D point correspondences [4]. We refer to the points used for establishing the 2D point correspondences as keypoints. These keypoints may belong to a marker, which is an object with a known shape that is either naturally occurring or artificially positioned in the scene. A regular pattern such as a chessboard is usually utilized [15]. A single marker is identified in the image by multiple independent keypoints that have a direct correspondence to its real shape, thus making a group of point correspondences. However, these correspondences are often noisy and they can introduce errors in the homography estimation. Although four keypoints are satisfactory, often a greater number of keypoints is used, allowing us to use optimization to minimize a suitable cost function [16,17]. Then, outlier removal becomes an important step, and algorithms such as RANSAC [18] are usually employed [16].

**Figure 1:**
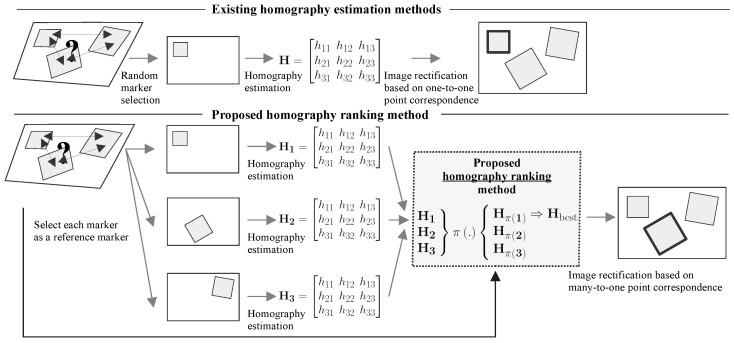
The difference between existing homography estimation methods and the proposed homography ranking method. In the presence of multiple markers without information about their relative positions in the world, existing approaches can only estimate isolated homographies without the ability to select the best one. Our method extends existing approaches by exploiting multiple markers to rank the isolated homographies.

Assume the presence of a sole marker at the scene. Even though the marker is distorted under perspective, the knowledge of its real shape makes it possible to compute the homography. When multiple copies of the same marker are visible but their positions in the world are unknown, the knowledge of the shape is not enough to incorporate all of the keypoints in the estimation. In the absence of position information, existing approaches for homography estimation based on point correspondences fail because the projection has to preserve the proportional positions. Thus, estimating the homography without knowing the ground-truth layout of the keypoints up to an arbitrary scale does not guarantee the correct result. Under the aforementioned constraints, existing methods can only generate an isolated homography for each marker based on the one-to-one point correspondence (see Figure 1). Each homography may be affected by different sources of noise, e.g., low resolution, blur, or keypoint detection. Thus, the outcome of rectification may vary. Additionally, in many practical applications, a single marker usually covers a small portion of the image, which increases susceptibility to noise. The trivial solution would be to use a bigger marker that covers the majority of the estimated plane in the image. However, this solution is often impractical. Furthermore, it is not possible to simply “merge” multiple isolated homographies together.

In this paper, we focus on exploiting information from multiple markers, i.e., multiple groups of point correspondences. We assume that the markers are placed on the same plane in the world over which we want to acquire the bird’s-eye view. We thus propose a homography ranking method that can incorporate information about multiple markers to select homography with the potentially minimal reprojection error. Therefore, the problem lies in determining which homography potentially achieves the best reprojection accuracy over the entire image. The proposed ranking method allows us to systematically select the best homography according to our score function when multiple choices are available. The outcome of our algorithm may serve as a recommendation for homography selection. We emphasize that we are not concerned with homography estimation itself. We only require point correspondences. Our algorithm can be implemented as an extension to existing approaches to sort a set of already estimated homographies according to their potential reprojection accuracy. We sidestep the need for the position information by constraining the markers’ shape. We assume that the markers in the world, while placed on the same plane, differ only in translation, rotation, and uniform scaling, i.e., a similarity transformation exists between them when viewed without perspective (see Section 3.1). The proposed method ranks homographies using our score function that computes a single value for each transformation matrix (see Section 3.2). The score value is used as a proxy to measure the reprojection “quality” of a specific homography over the whole image.

This work was motivated by a real-world application of generating a bird’s-eye view over a road from a video recording when we could not use a large marker to cover a sufficient portion of the road. Homography estimation based on a single small marker was inaccurate. Therefore, we tried to use multiple small markers and to measure their relative positions. However, their position measurements were highly noisy at best. Thus, the proposed method was used instead. Our method can also be adopted in a situation when a marker placed at various positions on the same planar surface is visible at different frames using a static camera. Stacking the frames onto each other yields a view with multiple markers.

Due to the exploitation of similarity transformations, the limitation of our approach is that it can only handle the projection from a distorted to the undistorted view of the target plane, not between various projective perspectives of the same plane. Therefore, it serves the removal of perspective distortion.

The experiments showed that the proposed method could systematically improve reprojection error by selecting the best homography according to our score function. We quantified the relative improvement in terms of reprojection accuracy ratio between the systematic homography selection and the baseline random selection. We used a random selection because the existing methods could not compare the “quality” of individual homographies and were therefore left with a random selection or some subjective rules. In practice, random selection would often be replaced by an educated guess. Without the loss of generality, the homographies in our tests were estimated using the implementation from the OpenCV [19] library. Thus, the main contribution of our work is as follows:

- The proposed method ranks (sorts) multiple homographies corresponding to individual markers placed on the same plane to select the “best” homography for rectification. Our method handles the absence of position information between markers in the world and builds on top of many-to-one point correspondences. The algorithm is an extension of existing methods since it works with already estimated homography matrices and does not alter them. This easy-to-implement extension is efficient, with a quadratic algorithmic complexity in the number of markers, which is usually very low.

The rest of the paper is organized as follows. The upcoming Section 2 contains an overview of related work. Then, in Section 3, we describe our proposed method. Section 4 is devoted to experiments and their evaluation. We summarize our conclusions in Section 5.

## 2. Related Work

To the best of our knowledge, there is no work related to the same narrow use case of homography transformation as what we deal with. In principle, our method can extend any homography estimation approach that satisfies the requirements. Therefore, in this section, we dissect various ways to estimate homographies and other works in which techniques that intersect with ours were employed.

### 2.1. Single Homography Estimation

Homography can be estimated using at least four point correspondences [3]. However, for this task, linear methods are sensitive to noise even if there are no outliers. To this end, many pre-processing steps have been developed. For example, normalizing each point set by translating the center of mass to the origin and by scaling appropriately [4]. Our goal is to tackle situations where point correspondences contain noise and thus outliers [20]. We mention markers as a demonstration of point correspondence. A paper that builds on fiducial markers and further homography refining is [21]. The authors discuss square and circular markers and propose a method to make extra adjustments to the initial homography estimate using point correspondences. Although we only focus on point correspondences, it is not the only way to identify a relationship between the observed marker and its ground-truth shape. For instance, circular markers pose new possibilities (e.g., the exploitation of vanishing lines) as well as challenges (e.g., ambiguity) for homography estimation. For a more detailed discussion, see [11].

If the system of equations formed by the point correspondences is overdetermined, then methods such as RANSAC [18] are used to separate inliers from outliers. Zhu et al. [22] developed an efficient algorithm to estimate the homography based on order-preserving constraints. In specific use cases, it is faster than RANSAC. As shown in [17], optimization-based approaches perform well with a large number of outliers.

Jawahar et al. [23] used object contours instead of point correspondences for homography estimation. Their algorithm started from affine transformation and iteratively advanced towards homography. We incorporate affine (similarity) transformations in our computations as well, but no iterative refining is involved. Chen et al. [24] proposed an iterative approach for homography estimation using point correspondences. Their contribution was to adopt a more reliable nonlinear geometric error rather than just an algebraic error. A thorough discussion of various computations of errors for homographies, including the geometric error, can be found in [25]. We employed the geometric error, too (see Section 3.2). Likewise, Li et al. [26] measured the reprojection error using the $l_{2}$-norm and showed that it is suitable for homography estimation.

Our core idea of assessing the quality of a homography matrix in terms of removing perspective distortion consists of measuring how accurately multiple objects with known shapes align with the expected shape after rectification. Song et al. [27] proposed a homography matrix evaluation method based on a geometric approach to increase the accuracy of aerial image matching. They assessed the transformation accuracy of a given homography by examining the shape of a transformed quadrangle. They matched the reference aerial image with the sensed image by iteratively refining the homography governed by the evaluation procedure. We do not have a reference image since we build on top of the reference object instead. One way to identify degenerate homographies is to compute the determinant or the condition number of the matrix [28]. Thus, the matrices can be assessed in terms of their “quality”, which is the purpose of our work. We exploited this property in one of our experiments concerning a homography optimization procedure we developed. See Appendix A for more details.

### 2.2. Multiple Homography Estimation

Bose et al. [29] presented a technique for a full affine and metric rectification of the ground plane by tracking moving objects. Their work is similar to ours in the exploitation of multiple instances of the same object at various places and the measurement of its properties. They estimated vanishing points based on non-parallel object trajectories to obtain the projective transformation and then used other geometric clues to deduce the affinity. On the other hand, several works use multiple planes to estimate a homography or to directly utilize multiple homographies.

A common strategy is to introduce additional constraints. In our work, we focus on one plane only with multiple already estimated homographies. Our constraints are related to similarity transformations. Taking advantage of multiple views of several planar surfaces may improve the single homography estimation since isolated plane homographies are compatible unless noise is present. In such a case, denoising constraints have to be introduced [30]. The work of [31] inspired [32] to tackle planar mapping and tracking by exploiting multiple frames and plane-induced homographies between them. Their system used nonlinear optimization. Chojnacki et al. [33] estimated multiple homographies linked together by consistency constraints. They estimated a set of homography matrices induced by multiple planes in the 3D scene between two views. Ruiz et al. [31] remarked that geometric constraints among multiple views could be used to recover a projective transformation. They proposed a simple 2D optimization method for the metric rectification of a single plane from several perspective images. In their setting, the camera(s) moved and a single object of interest was stationary. We either utilize a single moving object or multiple similar stationary objects in the scene under the assumption of being placed on the same planar surface.

Park et al. [34] proposed a panorama stitching method based on multiple frames using homography under the assumption of a static camera. The goal was to suppress the incorrect feature point extraction caused by time-varying noise to find the proper alignment parameters by estimating multiple homographies during a predetermined time intervals. To evaluate the accuracy, they employed metrics based on alignment distortion measurement. Cui [35] also highlighted the importance of homography to the segmentation of moving objects. Their proposed method allowed for using static and moving cameras by exploiting constraints based on multiple overlapped homographies.

Fraundorfer et al. [36] proposed a method that could recover scene planes of arbitrary position and orientation using multiple homographies and point correspondences. They employed iterative refining of the plane-induced homographies. We adopted a similar approach for evaluation. The authors also created a synthetic dataset with artificial fiducial markers in the scene. DeTone et al. [37] and Zhu et al. [22] adopted similar approaches to generate the synthetic homography-related dataset, too.

### 2.3. Deep Learning-Based Approaches

Some recent approaches make use of deep learning. In [37], they trained a neural network in a supervised manner to estimate the homography, whereas in [38], the homography was estimated from context using unsupervised learning. Deep learning favors use cases that pose a challenge for traditional approaches, e.g., dynamic scenes. Le et al. [39] proposed a deep learning-based approach to identify dynamic content in images and to estimate homography from coarse to fine using a multi-scale neural network trained in a multi-task fashion. They avoided iterative processes such as RANSAC. They also developed a suitable dataset as a response to the lack of available homography-related training data. Considering content awareness, the work of Zhao et al. [40] showed that deep learning boosts image stitching if the loss function considers image content. Homography estimation using deep learning was exploited in [41] to improve foreground segmentation. The majority of deep learning-based approaches still rely on four point correspondences. However, still, emerging works that estimate the homography directly instead of explicitly harnessing point correspondences show promising results, for instance [42].

## 3. Proposed Method

### 3.1. Preliminaries

**Figure 2:**
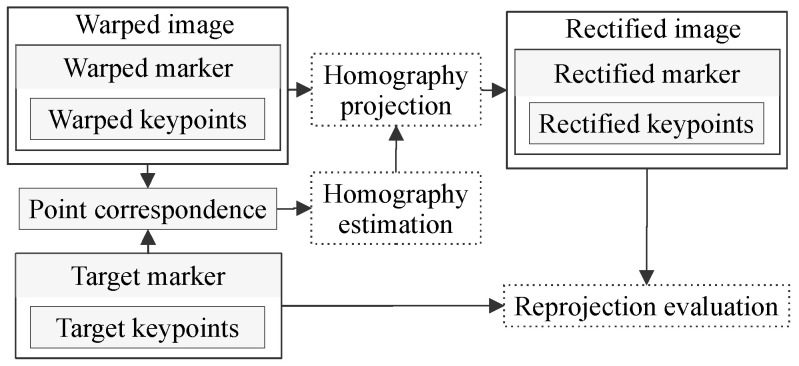
Visualization of relationships in our established terminology. The diagram also shows the hierarchical dependence of individual terms. Dotted elements represent processes with arrows denoting their input and output.

A marker is an object with a known, easy-to-detect shape. This object either naturally occurs or is artificially placed on the planar surface of the scene we want to produce a bird’s-eye view for, i.e., to remove perspective distortion. The marker contains keypoints, a set of distinct, independent, visual feature points, e.g., corners. Thechosen keypoints visible in the perspectively deformed image are called the warped keypoints. The set of the rectified keypoints in the desired image (not subjected to perspective distortion) is produced from the warped keypoints using the homography projection. The point correspondence is a relationship between the warped and the target keypoints, and it is used for homography estimation. Ideally, the rectified keypoints match the target keypoints. See Figure 2 for details.

The goal of homography estimation is to find the 3×3 homography matrix

(1) $$H=\left[\begin{array}{ccc} h_{11} & h_{12} & h_{13}\\ h_{21} & h_{22} & h_{23}\\ h_{31} & h_{32} & h_{33} \end{array}\right]$$

with 8 DoF [4]. A single vector $u^{T}=\left[u_{x},u_{y},1\right]$, representing a warped keypoint in homogeneous coordinates, is mapped onto the rectified keypoint ${\tilde{u}}^{T}=\left[{\tilde{u}}_{x},{\tilde{u}}_{y},1\right]$ by the homography H using the transformation $s\tilde{u}\approx Hu$, with *s* being the scale factor.

Without stating otherwise, a similarity transformation denotes a limited affine transformation with 4 DoF consisting of translation, rotation, and uniform scaling (Equation (5)). Let $K_{1}$ and $K_{2}$ be sets of feature keypoints belonging to objects $O_{1}$ and $O_{2}$. We say that objects $O_{1}$ and $O_{2}$ are similar if there exists a similarity transformation ψ such that $K_{1}=\psi \left(K_{2}\right)$ and $K_{2}=\psi^{-1}\left(K_{1}\right)$. For example, $O_{1}$ and $O_{2}$ may be rectangles of different sizes but with an identical aspect ratio.

Let *m* be the number of markers and *k* be the number of keypoints of each marker. Each *i*th marker is described by a 3×k matrix $W^{\left(i\right)}$ containing its warped keypoints as

(2) $$W^{\left(i\right)}=\left[\begin{array}{cccc} x_{1}^{\left(i\right)} & x_{2}^{\left(i\right)} & \ldots & x_{k}^{\left(i\right)}\\ y_{1}^{\left(i\right)} & y_{2}^{\left(i\right)} & \ldots & y_{k}^{\left(i\right)}\\ 1 & 1 & \ldots & 1 \end{array}\right],i=1,\ldots ,m.$$

The target keypoints are specified analogically by the 3×k matrix T. Only one specification is sufficient due to many-to-one correspondence. The ordering of keypoints needs to match the warped keypoints defined above. Thus,

(3) $$T=\left[\begin{array}{cccc} {\tilde{x}}_{1} & {\tilde{x}}_{2} & \ldots & {\tilde{x}}_{k}\\ {\tilde{y}}_{1} & {\tilde{y}}_{2} & \ldots & {\tilde{y}}_{k}\\ 1 & 1 & \ldots & 1 \end{array}\right],$$

with the point correspondence being

(4) $$x_{j}^{\left(i\right)}≃{\tilde{x}}_{j},y_{j}^{\left(i\right)}≃{\tilde{y}}_{j},i=1,\ldots ,m,j=1,\ldots ,k.$$

### 3.2. Homography Ranking Algorithm

**Figure 3:**
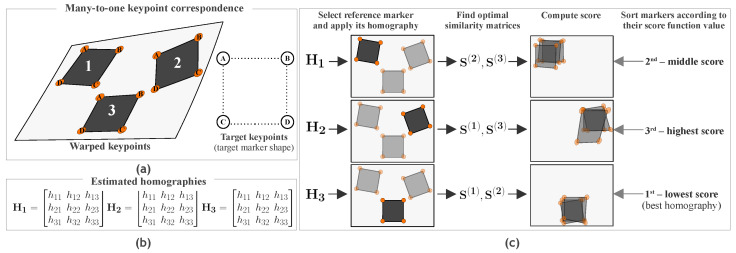
A system diagram describing the general idea behind our method. (**a**) The input consists of a many-to-one point correspondence specified by geometrically similar markers and information about the shape of the target marker. (**b**) We assume that the isolated homographies corresponding to each independent marker are provided on the input as well. (**c**) The algorithm processes each marker by applying its homography matrix to the image to produce a rectified image. Subsequently, it computes optimal similarity matrices corresponding to the auxiliary markers. The computation of the score function makes use of these transformations. The obtained score values then serve for comparison to rank (sort in ascending order) the homographies. The homography ranked first is considered the “best” candidate for the minimal reprojection error over the entire image.

Our method utilizes multiple similar markers (see Figure 3). The input is point correspondences and homographies estimated for each marker. Each marker is selected exactly once as a reference marker. All remaining markers are in the role of auxiliary markers. The reference marker’s homography is used to perform the perspective transformation to rectify all markers. To rank which reference markers’ homography yields the best reprojection, we exploit auxiliary markers. Auxiliary markers are subsequently mapped onto the target marker using similarity transformations (Equation (5)). We then convert the transformed keypoints to homogeneous coordinates and measure the reprojection error as the mean Euclidean distance between the rectified and the target keypoints (7). The aim is to minimize this quantity. The optimal similarity matrices are just auxiliary and redundant after the algorithm ends.

Let *r* be the index of the reference marker. The 3×3 matrices describing similarity transformations are contained in a set $S=\left\{S^{\left(i\right)}\;|\;i=1,\ldots ,m\right\}$, such that

(5) $$S^{\left(i\right)}=\left\{\begin{array}{c} \begin{array}{ccc} \; & \left[\begin{array}{ccc} 1 & 0 & 0\\ 0 & 1 & 0\\ 0 & 0 & 1 \end{array}\right] & \mathrm{if}\;i=r\\ \; & \left[\begin{array}{cc} R_{2\times 2}^{\left(i\right)} & T_{2\times 1}^{\left(i\right)}\\ 0_{1\times 2} & 1 \end{array}\right] & \mathrm{if}\;i\neq r \end{array} \end{array}\right.,$$

for i=1,…,m, where

(6) $$R_{2\times 2}^{\left(i\right)}=\left[\begin{array}{cc} s^{\left(i\right)}\cdot \cos\left(\theta^{\left(i\right)}\right) & -s^{\left(i\right)}\cdot \sin\left(\theta^{\left(i\right)}\right)\\ s^{\left(i\right)}\cdot \sin\left(\theta^{\left(i\right)}\right) & s^{\left(i\right)}\cdot \cos\left(\theta^{\left(i\right)}\right) \end{array}\right],\;T_{2\times 1}^{\left(i\right)}=\left[\begin{array}{c} t_{x}^{\left(i\right)}\\ t_{y}^{\left(i\right)} \end{array}\right].$$

This transformation (except for the identity) consists of 4 DoF: single rotation angle $\theta^{\left(i\right)}$, two *x* and *y* translation coefficients $t_{x}^{\left(i\right)}$, $t_{y}^{\left(i\right)}$, and a scale coefficient $s^{\left(i\right)}$. A full affine transformation with 6 DoF would be responsible for horizontal and vertical scales, shear and rotation, and *x* and *y* offsets [43]. The application of homography that rectifies an image produces a frontal plane that is related to the ground-truth plane by similarity transformation [3,44]. Thus, we do not include the shear and we only support uniform scaling (see Appendix A.1 for explanation).

Since all of the markers share the same planar surface, any homography has to provide a valid perspective projection, but all perspective projections are subjected to different noise. Our goal is to quantify which homography estimation provides the best perspective projection for the whole plane in the image. To do so, we propose a score function based on the aforementioned constraints. The score function computes a score for individual homographies in conjunction with estimated similarity matrices corresponding to auxiliary markers as

(7) $$F\left(H,S\right)=\frac{1}{m}\sum_{i=1}^{m}{∥h\left(S^{\left(i\right)}HW^{\left(i\right)}\right)-T∥}_{F},$$

where ${∥\cdot ∥}_{F}$ denotes the Frobenius norm. The function $h\left(\cdot \right)$ converts points to homogeneous coordinates as

(8) $$h\left(\left[\begin{array}{cccc} x_{1} & x_{2} & \ldots & x_{k}\\ y_{1} & y_{2} & \ldots & y_{k}\\ z_{1} & z_{2} & \ldots & z_{k} \end{array}\right]\right)=\left[\begin{array}{cccc} x_{1}\;/\;z_{1} & x_{2}\;/\;z_{2} & \ldots & x_{k}\;/\;z_{k}\\ y_{1}\;/\;z_{1} & y_{2}\;/\;z_{2} & \ldots & y_{k}\;/\;z_{k}\\ 1 & 1 & \ldots & 1 \end{array}\right].$$

Now, we describe the proposed Algorithm 1 for homography ranking. Assume a set of warped markers described by warped keypoints and a single target marker described by target keypoints. These objects are linked by a many-to-one point correspondence. Additionally, assume that homographies have been estimated for each marker in isolation. Our algorithm ascendingly ranks the input set of all pairs $\left(W^{\left(i\right)},T\right)$, i=1,…,m, by how well each *i*th marker preserves the target shape of all the markers in the image after removing the perspective distortion. This objective is measured by the score function defined in Equation (7). The algorithm evaluates all markers as candidates for the reference marker. In each iteration, it computes optimal similarity matrices for the auxiliary markers in the rectified plane, i.e., after applying the perspective projection induced by the current homography. The aim is to find a homography with a minimal score. The algorithmic complexity is quadratic in the number of markers; thus, $\Theta \left(m\left(m-1\right)+m\log_{2}\left(m\right)\right)≃\Theta \left(m^{2}\right)$.
**Algorithm 1** Homography ranking.  1:$\bar{H}\leftarrow \mathrm{array}\left[m\right]$               ▹ homographies  2:$s\leftarrow \mathrm{array}\left[m\right]$                     ▹ scores  3:**for** i←1,…,m **do**  4:    $\bar{H}\left[i\right]\leftarrow$ homography($W^{\left(i\right)}$, T)           ▹ perspective  5:    ${\bar{S}}^{\left(i\right)}\leftarrow I_{3\times 3}$  6:    $\bar{S}\leftarrow \left\{{\bar{S}}^{\left(i\right)}\right\}$              ▹ similarity matrices  7:    **for all** *j*: $\left\{1,\ldots ,m\right\}-\left\{i\right\}$ **do**  8:        ${\bar{S}}^{\left(j\right)}\leftarrow$ similarity($\bar{H}\left[i\right]\cdot W^{\left(j\right)}$, T)  9:        $\bar{S}\leftarrow \bar{S}\cup {\bar{S}}^{\left(j\right)}$10:    **end for**11:    $s\left[i\right]\leftarrow F\left(\bar{H}\left[i\right],\bar{S}\right)$              ▹ Equation (7)12:**end for**13:ω←argsort(s)                 ▹ indirect sort14:**return**$\;\bar{H},\omega$

It is important to note that the two functions used in this pseudocode to compute the homography and similarity matrices stand for arbitrary methods that produce the required transformations.

Our score function (7) is just a proxy for the reprojection error computed over the whole image. Since we utilize only a small subset of points from the entire image, which may be subjected to noise, the assumption that the “best” homography is the one our method ranks as first may not hold in every case. In very few cases, the marker that achieves the lowest score function value does indeed reconstruct the remaining markers the best but not the overall image. However, our experiments show that our method consistently preserves its performance under various conditions.

## 4. Experiments

We evaluated the proposed homography ranking algorithm in various conditions. We tested cases involving various similarity transformations applied to original markers as well as noisy point correspondence, e.g., errors in marker detection since these are the expected problems in real-world scenarios.

### 4.1. Implementation Details

Our proposed algorithm can extend any homography estimation method that exploits point correspondences. For demonstration, we adopted time-tested implementations from the OpenCV 4.4.0 library [19]. Each homography was estimated by the findHomography() function, which employs the DLT [45] algorithm for k=4 and the RANSAC [18] algorithm for k>4, where *k* is the size of the point correspondences set. Each optimal similarity transformation between two 2D point sets was estimated by the estimateAffinePartial2D(), which also utilizes RANSAC for robustness. We always used default parameters.

### 4.2. Dataset Creation

**Figure 4:**
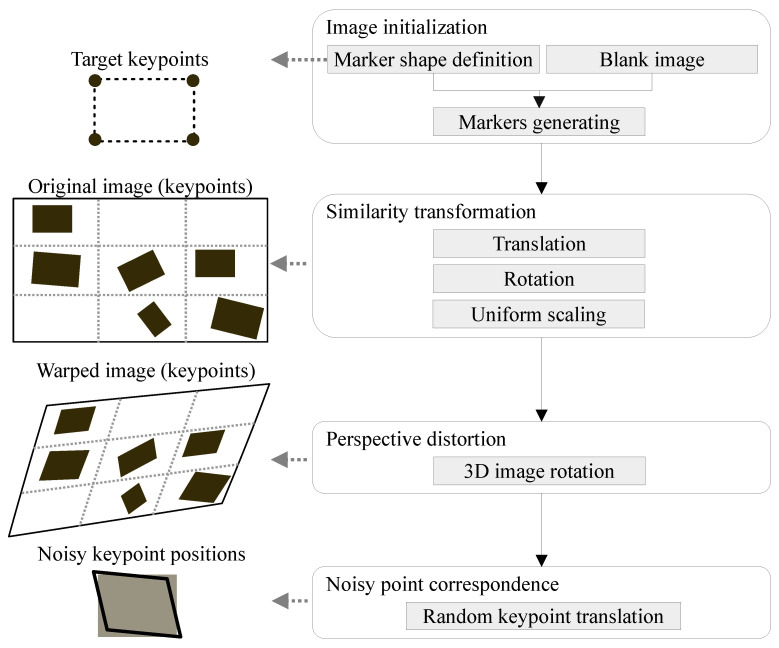
The description of how each one of the *t* test instances in a specific test scenario is created. The input is a blank w×h image over which *m* markers are initialized in a uniform grid, which produces the original marker keypoints. Depending on the test scenario, a particular subset of similarity transformations is applied to the entire image. Subsequently, warped keypoints are modified by random noise to simulate noisy point correspondence.

We created a synthetic dataset to simulate the presence of markers in the scene subjected to perspective distortion. Our experiments were based on a pixel-wise comparison of the reprojection error. The synthetic dataset covered multiple setups named the test scenarios. For each test scenario, we generated *t* different samples, which we refer to as test instances. We set t=1000. Table 1 contains description of the generated test scenarios. To create test instances (within test scenarios), we employed the procedures described below (see Figure 4).

We organized the creation of our dataset to allow for complete reproducibility of the reported results. Thanks to the synthetic nature of our data, fixing the seed for the used pseudo-random generator was sufficient. The source code for running the experiments is freely available (see the online Supplementary Materials at the end).

#### 4.2.1. Image Initialization

Each test instance was initialized as a blank 1024×768 image. This image served for *m* randomly generated copies of the same shape (marker) placed in a 3×3 grid, where 0<m≤9. We used a uniform border with 20% size of the corresponding side to prevent the generated shapes from reaching outside of the image. We experimented with a different number of markers. From the set of 3×3 possible anchors, we chose *m* randomly, onto which we placed the generated markers. We also studied the effect of 3, 5, 7, and 9 out of 9 possible markers, given that all of the similarity transformations and noise were applied. Regarding marker shapes, we tested squares or convex, equilateral polygons with a tight bounding box of size 100×100 pixels (covering approximately 1.3% of the image). However, other similar shapes could be used, too. Their centroids were evenly distributed over the image, whilst the grid cells served as anchors. We adopted random generators from a uniform probability distribution. These settings represented the default configuration. Subsequently, we applied further transformations to the generated markers and the image.

#### 4.2.2. Similarity Transformation

We showed the effect of similarity transformations before applying the perspective transformation. The translation and rotation demonstrate that markers could be positioned arbitrarily in a real environment provided that they shared the same planar surface. The change in scale showed that markers could be of different sizes.

To simulate a similarity transformation, we applied random rotation from the interval $\left[0,360\right)$ degrees with the origin in the marker center. Then, we generated a random coordinate shift from interval $\left[-20,20\right]$ pixels for translation in the *x* and *y* directions. However, an identical translation had to be applied to the entire marker to prevent distortion. Then, uniform scaling was performed with the origin in the marker center with a scale factor randomly generated from the interval $\left[0.8,1.5\right]$. Due to this range, a ratio of the marker to image area ranged from 1.0% to 1.9%.

#### 4.2.3. Perspective Distortion

We simulated a 3D rotation of an image around its center to represent a change in perspective on the plane that contained several markers. We rotated the image around its center in the *x*, *y*, and *z* axis by a random angle from interval $\left[-20,20\right]$ degrees to achieve a change in perspective. The original keypoints were transformed along with the entire image, producing the warped keypoints.

#### 4.2.4. Noisy Point Correspondence

To simulate a noisy point correspondence, we applied a random noise (translation) to each *x* and *y* coordinate of the warped keypoints from the interval $\left[-2,2\right]$ pixels. At this stage, each keypoint was modified in isolation to achieve the distortive effect. Thanks to the perspective deformation, the generated random shift represented different levels of noise depending on how much the image had been warped. This step imitated errors in the marker detection, leading to a noisy point correspondence.

### 4.3. Evaluation Methodology

#### 4.3.1. Error Computation

We evaluated the accuracy of our method by measuring the reprojection error using the Euclidean distance between the original and the rectified pixel positions. To obtain an error over the entire image, we computed the error for each pixel. Let *w* and *h* be the width and height of the image, respectively. The 3D rotation of a point in the image around the image center that produces perspective distortion is represented by $\varphi \left(\cdot \right)$. Let $g_{i,j}^{T}=\left[j,i,1\right]$ be the original (ground-truth) pixel position at the *i*th row and *j*th column, and let $w_{i,j}=\varphi \left(g_{i,j}\right)$ be the analogically defined warped pixel position, for i=1,…,h,j=1,…,w. We then compute the 2D reprojection error grid (a h×w matrix) for the given homography H as

(9) $$\xi_{wh}=\left[\begin{array}{ccc} e\left(w_{1,1},g_{1,1}\right) & \ldots & e\left(w_{1,w},g_{1,w}\right)\\ \ldots & \ldots & \ldots \\ e\left(w_{h,1},g_{h,1}\right) & \ldots & e\left(w_{h,w},g_{h,w}\right) \end{array}\right],$$

where

(10) $$e\left(w,g\right)={∥Hw-g∥}_{2}.$$

To express the reprojection error as a single number for the whole image, we adopted an arithmetic mean of all the values in the error grid above, so

(11) $$\xi_{\mathrm{reproj}}=\frac{1}{wh}\sum_{i=1}^{h}\sum_{j=1}^{w}e\left(w_{i,j},g_{i,j}\right).$$

#### 4.3.2. Evaluation Algorithm

On the input, we have *m* markers (Section 4.2) and thus an *m*-to-1 point correspondence. Each marker provides its unique homography. Our goal is to quantify the relative improvement in the reprojection error over the baseline when the *k*th ranked homography is used for rectification. Even though we are primarily concerned only with the single, top-performing homography, we evaluate the entire ranking to demonstrate stable behavior.

We evaluated our homography ranking in terms of reprojection error improvements against the existing approaches based on the isolated homography estimation represented by OpenCV [19] implementation. Since our method provides a ranking, we compare our performance against a random marker selection based on uniform probability distribution. We refer to this performance as the “baseline”, an unbiased marker selection. To obtain the aforementioned baseline, we evaluated the reprojection error (11) for each marker in isolation and computed the arithmetic mean of these values. When we executed our proposed algorithm, we obtained the full ordering of markers by their score value computed using the proposed criterion (7). We expected that, if the first marker is used to rectify the image, then the reprojection error is minimal (and lower than the baseline error). If any subsequent marker in the given order is used instead, the reprojection error increases.

We computed the relative improvement in % for each *k*th homography according to the baseline performance. Each test scenario was evaluated separately. For each test instance, we obtained a *k*-dimensional vector, where its elements represented percentual improvement at each *k*th position. We represented our data as a t×k matrix, where *t* was the number of test instances. We treated each column separately to compute the statistics. Our evaluation algorithm is described in Algorithm 2. For simplicity, we show an evaluation of a single instance.
**Algorithm 2** Evaluation algorithm.  1:$\bar{H},\omega \leftarrow$rankhomographies( )         ▹ Algorithm 1  2:$e_{b}\leftarrow 0$                    ▹ baseline  3:$e\leftarrow \mathrm{array}\left[m\right]$            ▹ reprojection errors  4:$p\leftarrow \mathrm{array}\left[m\right]$          ▹ relative improvements  5:**for** i←1,…,m **do**  6:    $e\left[i\right]\leftarrow$$\xi_{\mathrm{reproj}}$               ▹ Equation (11)  7:    $e_{b}\leftarrow e_{b}+e\left[i\right]$  8:**end for**  9:$e_{b}\leftarrow e_{b}/m$           ▹ mean reprojection error10:**for** i←1,…,m **do**11:    $k\leftarrow \omega \left[i\right]$           ▹ position of *i*-th best homography12:    $p\left[i\right]\leftarrow \left(e_{b}-e\left[k\right]\right)/e_{b}$         ▹ relative improvement13:**end for**14:**return **p

### 4.4. Results

**Figure 5:**
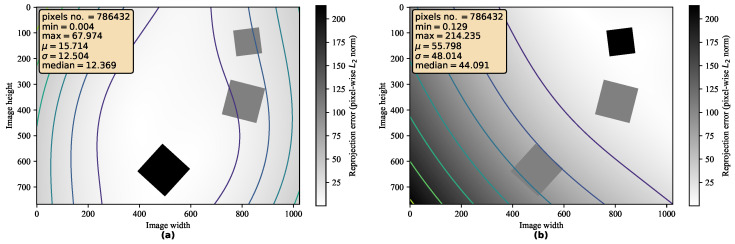
Distribution of pixel-wise reprojection error. The heat map together with corresponding contours demonstrate the varying distance between the ground truth and rectified pixel position after removing the perspective distortion. The bold square represents the reference marker. We show the result of (**a**) the “best” marker and (**b**) the “worst” marker. This test scenario includes all similarity transformations as well as noise in point correspondence.

Figure 5 shows how the reprojection error varies with respect to the marker position. We can see that the marker position can be deduced by looking at the heatmap representing the pixel-wise reprojection error over the image. The transformation achieves the best accuracy in the marker neighborhood and steadily decreases for more distant pixels. However, not all markers are subjected to the same pattern of error variation. This observation was the core motivation for our solution. We aim to choose the marker that minimizes the pixel-wise reprojection error within the region of the image that is as broad as possible. That is why we evaluate our method by computing the reprojection error over each pixel, not just the keypoints.

**Figure 6:**
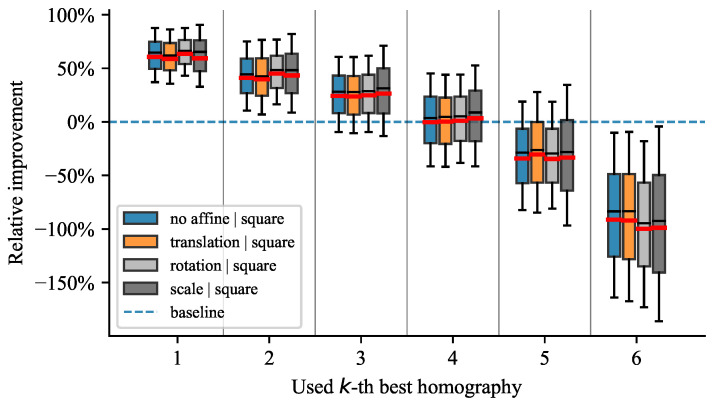
Influence of similarity transformation on the reprojection error.

**Figure 7:**
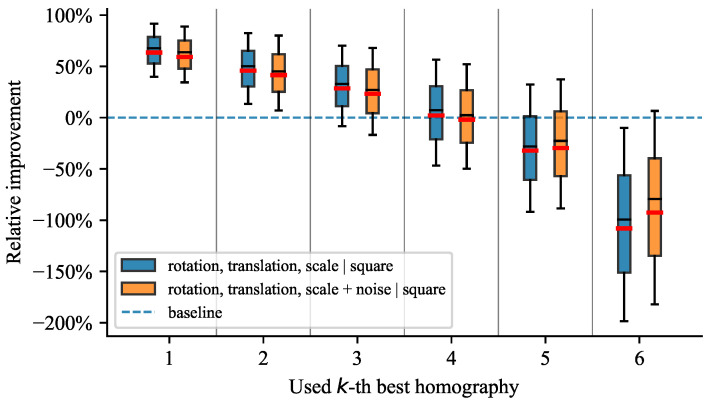
Influence of noise applied to the warped keypoints representing a noisy point correspondence.

**Figure 8:**
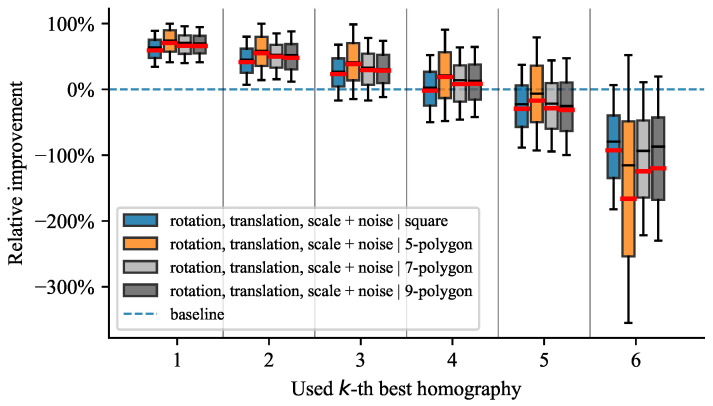
Results for different marker shapes.

**Figure 9:**
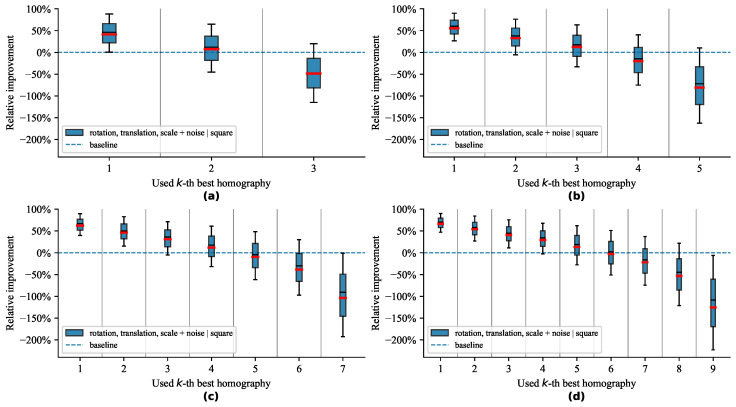
Influence of a different number of markers on reprojection error. We experimented with (**a**) three, (**b**) five, (**c**) seven, and (**d**) nine markers.

All tested scenarios depict similar trends, as shown on the plots in Figure 6, Figure 7, Figure 8 and Figure 9. The box plots extend from the lower to upper quartile values, with the thin and thick lines representing the median and mean, respectively. The plots discussed further show relative improvements over the baseline OpenCV [19] method. We evaluated relative improvements for the sake of interpretability. For better comprehension, we present Table 1. It contains individual test scenarios and their corresponding top performances in percents. Conversely, the reprojection error in absolute terms is difficult to interpret without additional context. Nevertheless, to highlight the differences in reprojection errors, we also provide absolute values in Table 1. The presence of noise shifted the errors by multiple magnitudes but still preserved the pattern of distribution.

#### 4.4.1. Influence of Similarity Transformations

In this test scenario, we tested each allowed similarity transformation in isolation, i.e., translation, rotation, and uniform scaling. Figure 6 demonstrates that the relative improvement was circa equal in all situations. Moreover, we show that the proposed method is practically invariant to similarity transformations allowing the markers to be in arbitrary positions in a plane. When all similarity transformations were utilized, our method performed even better, showing its stability and robustness.

#### 4.4.2. Influence of Noise

In Figure 7, we can see the effect of a noisy point correspondence that simulated an inaccurate keypoint detection. The ranking method preserved the trend of the relative improvement in the presence of noise. The absolute reprojection error demonstrated that, unless noise was present, the errors varied on sub-pixel levels, so they were practically zero.

#### 4.4.3. Influence of Variable Shapes

We expected that the relative improvement of our method should be invariant to variable shapes as long as they were similar. Figure 8 demonstrates that, with an increasing number of keypoints, our method consistently preserved its capabilities. Introducing more complicated shapes than just rectangles did not exacerbate the outcome of the algorithm.

#### 4.4.4. Influence of Number of Markers

We tested a variable number of markers to demonstrate that our method preserved its improvement. Figure 9 shows that, the greater the set of markers, the better the relative improvement of our method. Even when we used just three markers, the proposed method achieved a 46.91% median relative improvement. While it is beneficial to use a larger number of markers, we believe that the improvement we can obtain from an increasing number of markers has a logarithmic trend. On the extreme side, if we used only one marker, there would be no improvement since there would be only one homography to choose from.

## 5. Conclusions

In this paper, we proposed a method that builds on top of existing approaches for homography estimation that utilize point correspondences. Our method systematically ranks a set of homography matrices according to our proposed score function. Each homography in this set belongs to a specific marker. These markers are objects of known shape either naturally occurring or purposely placed in the scene.

This method is based on three assumptions. The first is that the markers are geometrically similar, i.e., they differ only in translation, rotation, and uniform scale in the real world. The second is that the shape of at least one of them is known. The third is that these markers are placed on the same planar surface in the scene. Our approach shows a way to relate all of the markers to each other in a single score function without knowing their relative positions in the scene. Our method only handles transformation from a distorted to the undistorted view of the target plane. Thus, it serves the removal of perspective distortion.

We exploited the properties of homography and similarity transformations and expressed them in a single score function. This function stands at the core of our contribution. Its value is used as a proxy to rank homographies according to their reprojection error over the entire image using only markers’ keypoints. The usual use case would be to select the homography with the lowest score, i.e., the highest-ranked matrix, to perform the image rectification.

We demonstrated that the proposed solution is robust in the presence of noise in the point correspondences. These correspondences can be either algorithmically found using feature-matching algorithms (e.g., SIFT [46] and SURF [47]) or annotated manually. However, even human annotations are often inaccurate. We also showed the robustness of our method to a varying number of markers and a change in shape.

All of our test scenarios demonstrated the following trend. On average, the homography with the highest score improved the relative performance to the baseline performance the most (both median and mean above 60%). The lowest-ranked homography often led to a lot worse performance (median and mean around −90%). These values varied slightly across different setups. The shape and number of markers had the greatest influence. All of the improvements in between steadily decreased and reached 0% improvement at around 2/3m, where *m* is the number of markers. A general claim is that the first half of ranked homographies yields a better reprojection compared with the baseline on average. The baseline performance was given by an average OpenCV [19] reprojection error under the assumption of no prior preference of specific markers, hence the random marker selection.

Our algorithm is invariant to the underlying homography estimation method. It can thus serve as an extension to approaches that handle point correspondences, either as part of run time or a post-processing stage. Moreover, it is computationally very efficient, as it scales well with a quadratic complexity $\Theta \left(m^{2}\right)$.

## Figures and Tables

**Table 1:** Description of the test scenarios in our synthetic dataset with corresponding settings and results for the top-ranked homography. One row represents one test scenario. Four visually separated groups (from top to bottom) are related to experiments shown in Figures 6–9.

Shape	Markers	Transl.	Rotation	Scale	Noise	Top Relative Improvement			Top Absolute Improvement		
						Median	Mean	Stdev	Median	Mean	Stdev
square	6	no	no	no	no	62.80%	59.63%	19.64%	0.00029	0.00030	0.00014
square	6	yes	no	no	no	62.65%	59.00%	19.72%	0.00028	0.00029	0.00013
square	6	no	yes	no	no	66.42%	63.17%	19.11%	0.00041	0.00043	0.00020
square	6	no	no	yes	no	63.38%	58.51%	23.97%	0.00024	0.00025	0.00015
square	6	yes	yes	yes	no	67.82%	63.66%	20.30%	0.00035	0.00037	0.00019
square	6	yes	yes	yes	yes	64.11%	59.26%	22.12%	22.07813	24.31773	15.00850
5-poly	6	yes	yes	yes	yes	74.67%	71.19%	21.98%	69.55532	336.26534	685.74274
7-poly	6	yes	yes	yes	yes	71.02%	65.63%	22.99%	46.79390	135.65737	395.75257
9-poly	6	yes	yes	yes	yes	68.97%	65.57%	21.98%	44.97627	115.12189	309.27201
square	3	yes	yes	yes	yes	46.91%	41.36%	31.58%	14.77504	18.11548	20.67457
square	5	yes	yes	yes	yes	59.03%	53.91%	24.56%	19.76285	22.53333	16.00804
square	7	yes	yes	yes	yes	66.19%	62.41%	19.98%	23.87681	27.13637	32.28533
square	9	yes	yes	yes	yes	69.86%	66.09%	18.18%	25.66452	26.68378	11.69754

## Data Availability

Our study involved only synthetic data generated at runtime during the execution of our experiments. We provided the link to the source code of our entire pipeline in Supplementary Materials above.

## Supplementary Materials

The following are available online at https://github.com/mondrasovic/homography_ranking, accessed on 26 August 2021.

## Appendix A

### Appendix A.1. Method Details

In this part, we provide more mathematical details concerning our score function defined in Equation (7). It is based on the idea that the knowledge of the homography allows us to remove the perspective distortion and to then apply any similarity transformation, i.e., translation, rotation, and uniform scaling [44]. We can transform the perspectively distorted view of the plane into a rectified form where parallelism and the ratio of lengths and angles are preserved.

A homography is a perspective projection between different views of a planar surface; hence, a 3D-to-2D transformation is reduced to a 2D-to-2D transformation. However, if the planar surface is frontal, i.e., it is perpendicular to the optical axis of the camera, then homography reduces to similarity transformation. Consequently, if we rectify the image, then the only allowed transformations are similarity transformations that we exploit in the computation of our score function. Moreover, this also explains why the shear has to be omitted.

Let $p^{T}=\left[x,y,z,1\right]$ be an arbitrary point that lies on a frontal plane given in world coordinates. Suppose that the optical axis of the camera is represented by the *Z*-axis. Then, $p^{T}=\left[x,y,0,1\right]$. A projection of the point p onto a point ${\left[\tilde{u},\tilde{v},1\right]}^{T}$ in the image plane using the pinhole camera model is given by

(A1) $$\left[\begin{array}{c} \tilde{u}\\ \tilde{v}\\ 1 \end{array}\right]\approx \left[\begin{array}{cccc} f & 0 & 0 & 0\\ 0 & f & 0 & 0\\ 0 & 0 & 1 & 0 \end{array}\right]\left[\begin{array}{cccc} r_{11} & r_{12} & r_{13} & t_{x}\\ r_{21} & r_{22} & r_{23} & t_{y}\\ r_{31} & r_{32} & r_{33} & t_{z}\\ 0 & 0 & 0 & 1 \end{array}\right]\left[\begin{array}{c} x\\ y\\ 0\\ 1 \end{array}\right],$$

which simplifies to

(A2) $$\left[\begin{array}{c} \tilde{u}\\ \tilde{v}\\ 1 \end{array}\right]\approx \left[\begin{array}{ccc} fr_{11} & fr_{12} & ft_{x}\\ fr_{21} & fr_{22} & ft_{y}\\ r_{31} & r_{32} & t_{z} \end{array}\right]\left[\begin{array}{c} x\\ y\\ 1 \end{array}\right],$$

where *f* is the scale (focal length); $r_{ij}$ for i,j=1,…,3 specifies the rotation; and $t_{x}$, $t_{y}$, and $t_{z}$ denote the translation. The 3D rotation is reduced to a 2D rotation:

(A3) $$\left[\begin{array}{c} \tilde{u}\\ \tilde{v}\\ 1 \end{array}\right]\approx \left[\begin{array}{ccc} f\cos\left(\theta \right) & -f\sin\left(\theta \right) & ft_{x}\\ f\sin\left(\theta \right) & f\cos\left(\theta \right) & ft_{y}\\ 0 & 0 & t_{z} \end{array}\right]\left[\begin{array}{c} x\\ y\\ 1 \end{array}\right],$$

yielding a similarity transformation. Therefore, our score function needs to encompass the same similarity transformations that could affect the objects on the planar surface in the real world.

Consider the following hierarchy of transformations: similarity, affine, and projective. A projective transformation can be decomposed into a chain of transformations, where each matrix is given by a transformation that is higher in the hierarchy than the previous one [3,29]. Specifically, a homography H may be decomposed into similarity, affinity, and projectivity as follows:

(A4) $$H=H_{S}H_{A}H_{P}=\left[\begin{array}{cc} sR & t\\ 0^{T} & 1 \end{array}\right]\left[\begin{array}{cc} K & 0\\ 0^{T} & 1 \end{array}\right]\left[\begin{array}{cc} I & 0\\ v^{T} & v \end{array}\right],$$

such that v≠0 [3].

Our score function exploits the transformation of the plane into a frontal plane that is related to the ground-truth plane by similarity. It uses a homography followed by a similarity transformation to quantify the reprojection error for keypoint rectification. Besides the empirical evidence, the transformation using a homography followed by a similarity transformation has a simple theoretical justification.

The similarity matrix is a special case of the homography matrix. According to Equation (A4), it may be decomposed as

(A5) $$S=H_{S}^{\left(S\right)}H_{A}^{\left(S\right)}H_{P}^{\left(S\right)}=H_{S}^{\left(S\right)}\cdot I\cdot I.$$

A composition of linear transformations gives us

(A6) $$H^{\prime }=SH=H_{S}^{\left(S\right)}H=\left(H_{S}^{\left(S\right)}H_{S}\right)H_{A}H_{P}=H_{S}^{\prime }H_{A}H_{P},$$

which is a homography transformation, too.

### Appendix A.2. Joint Optimization

Our score function defined in (7) evaluates the reprojection error of multiple markers when a similarity transformation is applied after rectification. Given the homography ranking approach introduced in Algorithm 1, we observe that the required transformation matrices can be either retrieved from the input or estimated in isolation at run time. We attempted to perform a joint optimization where the homography matrix H, together with the set of similarity matrices S, were estimated simultaneously. Thus, all DoFs were treated as decision variables.

Our optimization objective function was the unmodified score function from Equation (7). We used the L-BFGS algorithm implemented in PyTorch [48], which is an iterative method for solving unconstrained nonlinear optimization problems [49] with good performance for non-smooth optimizations [26,50]. It utilizes the first-order gradients and the estimated inverse Hessian matrix. We obtained the gradients using automatic differentiation [48]. The initial estimates for the homography and the similarity matrices were obtained using the OpenCV [19] implementations (see Section 4.1).

The optimization was unstable because the algorithm was allowed to update all of the unknowns simultaneously, which led to exploding gradients. We thus split the optimization into two parts: isolated refining of the homography while having the coefficients for similarity transformations frozen. Then, the roles changed, and similarity transformations were refined while keeping the homography untouched. This sometimes resulted in degenerate homography matrices, i.e., their determinant was negative or very close to zero [28]. We then expanded the objective by adding a penalty term for the determinant value. It stabilized the optimization, improved convergence, and produced usable homographies. However, this joint optimization brought either no or just a minor improvement (approximately 3%) in the reprojection error. Moreover, the computational overhead was substantial. We believe that, since the proposed score function incorporates all of the markers, it brings the greatest improvement by itself. Further refinement of the involved transformations will probably have diminishing returns.
